# The process, outcomes and context of the sanitation change induced by the Swachh Bharat Mission in rural Jharkhand, India

**DOI:** 10.1186/s12889-024-18388-y

**Published:** 2024-04-12

**Authors:** Josef Novotný, Radhika Borde, František Ficek, Anant Kumar

**Affiliations:** 1 Department of Social Geography and Regional Development, Faculty of Science, Charles University, Albertov 6,, Prague,, Czechia; 2 School of Geography, University of Leeds, Leeds, UK; 3 Xavier Institute of Social Service, Purulia Road, 834001 Ranchi, Jharkhand India

**Keywords:** Environmental health, Sanitation, Swachh Bharat Mission, Jharkhand

## Abstract

**Background:**

The Indian Swachh Bharat Mission (SBM) was launched in 2014 with the goal to make India open defecation (OD) free by October 2019. Although it is known that the ambitious goal was not achieved, the nature of the sanitation change brought about by the SBM in different parts of India is poorly understood. One reason is a dearth of case studies that would shed light on the performance of the SBM simultaneously across its different domains. This article provides an example of such study. Employing a Process, Outcomes, Context approach, the objective is to understand the process and outcomes of the SBM-induced sanitation change in a specific context of rural Jharkhand.

**Methods:**

The study utilizes data collected through field research conducted in the rural areas of Ranchi district, Jharkhand, a state in east-central India. This data was obtained via repeated cross-sectional household surveys conducted at the beginning and at the end of the SBM, supplemented by key informant interviews with SBM stakeholders.

**Findings:**

We identified political support of SBM implementation and its acceptance amongst the population. Female community workers became key agents of SBM implementation at local level. The SBM increased toilet coverage in the study area from 15% to 85% and lowered the OD rate from 93% to 26%. It substantially reduced structural inequalities in access to toilets, furthered social sanitation norms, improved some of the attitudes towards toilet use, but impacted less on hygiene and sanitation knowledge. The implementation mainly concentrated on the construction of subsidized toilets but less on improving public understanding of safe sanitation practices.

**Conclusions:**

Although the SBM reduced sanitation inequalities in access to toilets in the study area, the behaviour change component was underplayed, focusing more on spreading normative sanitation messages and less on public education. Sustainability of the observed sanitation change remains a key question for the future. This article calls for more systematic production of geographically situated knowledge on the performance of sanitation interventions.

**Supplementary Information:**

The online version contains supplementary material available at 10.1186/s12889-024-18388-y.

## Introduction

Unsafe sanitation still accounts for a notable share of the global disease burden, especially amongst children in low- and middle-income countries [1, 2]. This is particularly true for India [3, 4], a country with a major influence on global progress towards safe sanitation [5]. The Indian government has repeatedly attempted to improve the unsatisfactory sanitation situation in the country by several large-scale sanitation campaigns such as the National Water Supply and Sanitation Programme (1954), the Central Rural Sanitation Programme (1986), the Total Sanitation Campaign (1999–2012), the Nirmal Bharat Abhiyan (2012–2014). However, these earlier schemes did not yield satisfactory results [6]. In 2014, the Swachh Bharat Mission (SBM) was launched in India as the largest ever sanitation programme with the declared goal to eliminate open defecation (OD) by October 2019. As a flagship initiative of the Indian government, the SBM gained unprecedented political support and was heavily promoted. Its implementation has also been portrayed as comparatively successful [7–9], though this is not without contestation [10–12].

The official SBM records state that toilet coverage in rural India increased from 39% in 2014 to almost 100% in October 2019 by constructing 103 million toilets and initiating toilet use amongst 550 million people across rural India [13]. However, the most recent round of the National Family Health Survey (NFHS-5) uncovered that only 65% of people in rural India used improved toilets in 2019–2020 [14]. Although this is a considerable increase from the corresponding figure of 37% reported by the 2015-16 NFHS-4 [15], a lot remains to be done to eliminate OD in India. It also indicates that official SBM records might have been inflated and/or that a substantial part of the toilets constructed under the SBM have not been used [16].

All-India estimates hide significant regional disparities in sanitation rates [12, 14, 17–20] which may reflect differences in the pre-SBM sanitation rates but may also be related to variation in SBM implementation and performance across India. The focus of this article is on rural Jharkhand, which is one of the Indian states with the most alarming sanitation situation before SBM implementation [21, 22]. The toilet coverage was only 8% based on the Census of 2011. According to the NFHS-4 survey caried out in Jharkhand in 2016, 12% of rural households used improved sanitation facilities, while the share increased to 51% as per NFHS-5 2020/21 survey [14].

Besides the development in aggregate sanitation rates, the nature of sanitation change induced by SBM in specific contexts has not been adequately understood. Published research on the SBM contains a set of studies that tested specific experimental behaviour-change adaptations of the SBM implemented in a few regions across India such as in Odisha [23–26], Karnataka [27], Bihar [28], Gujarat [29, 30] or Punjab [31]. Although interesting, these studies shed less light on the performance of the SBM in “ordinary” settings (i.e., the majority of India, where such experimental adaptations were not implemented). Moreover, they typically examined only one or a few outcomes or selected thematic areas and often considered only part of the SBM programme period. The same holds for other research on the SBM focused on the change in main sanitation outcomes such as toilet coverage and use [12, 18–20, 32] or on specific aspects of the SBM such as its gender dimension [33–36], psychosocial stress or implementation and attitudes towards it [7, 10, 37].

**Fig. 1:**
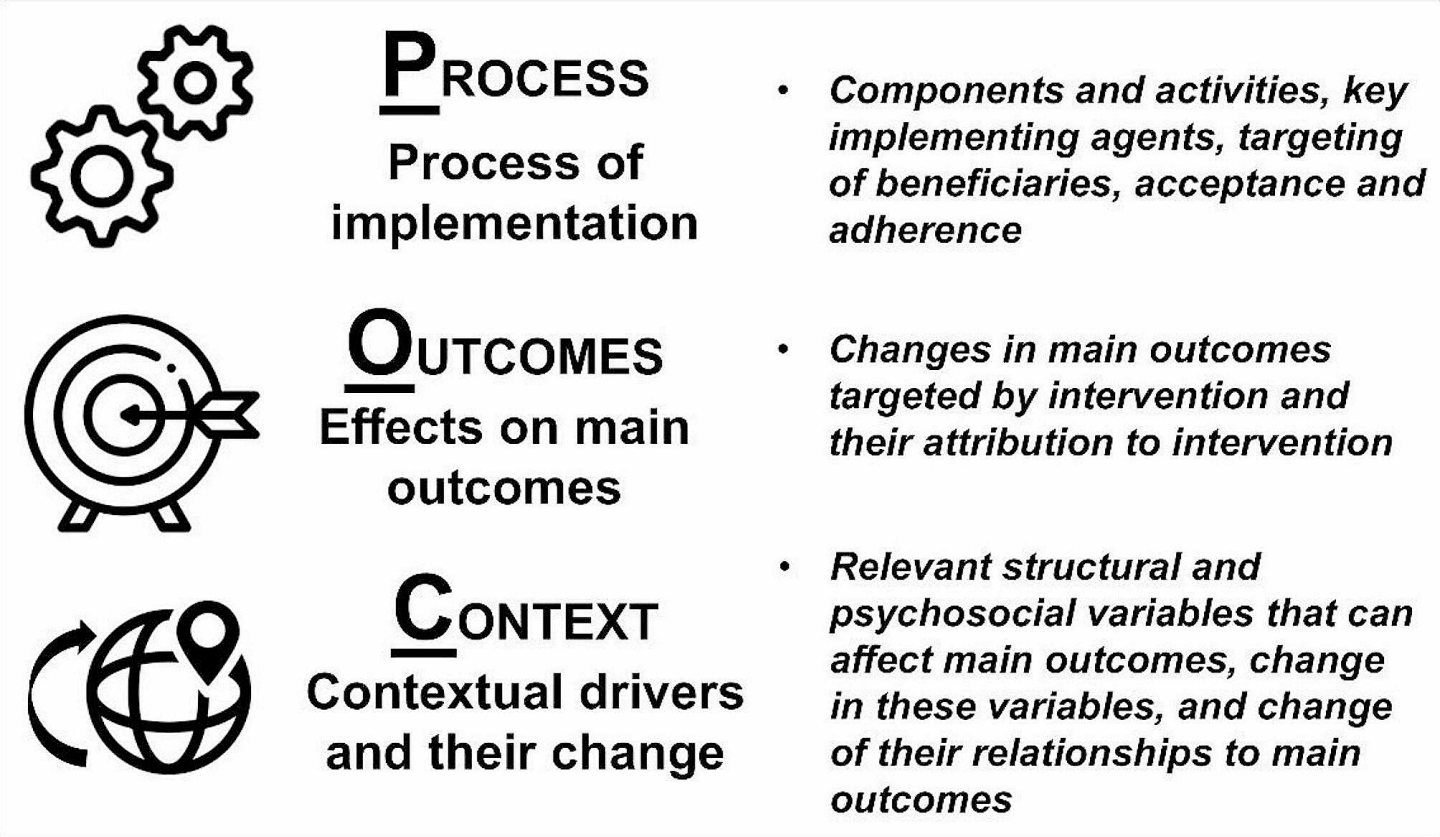
POC heuristics adopted in this study

Unlike in the above-mentioned literature on the performance of SBM, we seek to apply a less reductionist and more holistic view on SBM-induced sanitation change. Our general objective is to understand this change in the study area across its multiple domains. To achieve this goal, we follow a simple heuristic outlined in Fig. 1 referred to as the Process, Outcomes, Context (POC) approach, which leans towards a realist evaluation perspective [38, 39]. First, we scrutinize the process of the SBM implementation in the study area, focusing primarily on its grassroots-level implementation, key agents, and beneficiaries of the SBM. Second, we examine the changes in sanitation conditions in the study area, including the extent to which they can be attributed to the implementation of the SBM, particularly its impacts on the main outcomes of toilet coverage and use. In the third step, we analyse the role of local contextual drivers, focusing on the measurable situational variables of individuals, households, or their communities that can influence the targeted main sanitation outcomes. We acknowledge that the role of these contextual variables can change during and/or due to SBM implementation. Therefore, comparing the roles played by these variables with respect to the main sanitation outcomes before and after SBM implementation is a key aspect of our analysis in the third step.

To define these situational variables that measure local context, we draw on the basic assumption that successful sanitation interventions hinge on addressing both structural constraints and psychosocial antecedents for safe sanitation. Accordingly, following a previous study [40], we consider two types of situational variables. The first type, referred to as structural variables, includes objective characteristics, such as age, income, general education, social group, religious affiliation, etc., that help us assess the presence or absence of structural sanitation inequalities in the study area. The second type comprises subjective sanitation-related psychosocial variables, such as sanitation and hygiene knowledge, attitudes, risk perceptions, and perceived norms around sanitation. These variables capture the local situation with respect to the presence or absence of behavioural antecedents for safe sanitation practices. The comparison of their roles before and after SBM implementation is crucial as it addresses the underlying psychosocial mechanisms of sanitation change [41, 42].

In summary, the overarching goal of understanding sanitation change induced by the SBM in rural Jharkhand is achieved by addressing the following specific research questions:

1. How was the SBM implemented at the grassroots level in the study area, and how was it received by the local population?
2. What impact did the SBM implementation have on sanitation conditions, specifically the accessibility and use of functional toilets in the study area?

3a. Did the SBM reduce structural sanitation inequalities in the study area?

3b. How did the SBM influence the psychosocial antecedents of hygienic sanitation practices?

Our case study examines a rural part of Ranchi district in which 85% of households practiced OD as per Census 2011. It is a culturally diverse region within a Fifth Schedule (tribal majority) area, which is specific in relation to land rights, identity politics, gender dynamics and environmental knowledge/attitudes [43–45]. This regional specificity may have shaped sanitation attitudes and behaviours as well as the implementation of SBM [40, 46].

This study is based on data collected by a mix of methods. The main data come from two cross-sectional household surveys conducted in the same set of villages, but not the same set of households, of Ranchi district at the beginning of SBM implementation in 2016 (*N* = 499) and after its end in 2019 (*N* = 871). This is supplemented by qualitative information from semi-structured interviews and focus group discussions with SBM implementers (*N* = 71).

Revolving around complex human-environment interactions, sanitation change tends to be highly context-dependent [47–49]. Understanding the underlying processes and contextual drivers is thus required for interpreting and generalising evidence on the change in main sanitation outcomes such as toilet coverage and use. Realistically, this task can be better facilitated by adequately scoped local case studies based on a mix of observational methods rather than through narrowly focused experimental methods [50–53]. With this article, we aim to present a case study that offers a nuanced understanding of SBM performance in a given context.

## Data and methods

### Data

**Fig. 2:**
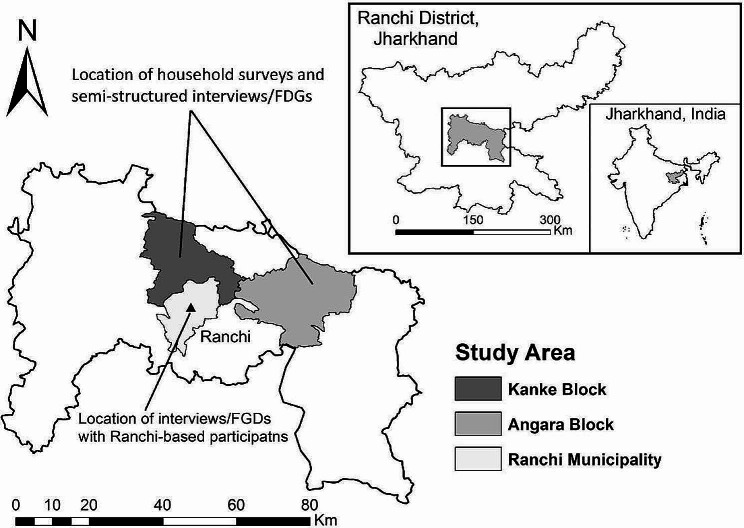
Location of the study area and data collection

The research site comprises 12 Gram panchayats in Angara and Kanke blocks of Ranchi district of Jharkhand (Fig. 2). The panchayats were selected purposely for practical feasibility reasons in terms of the support from a local NGO. It helped us in securing necessary permissions (both formal and informal) in the initial phase of the research but played no role in the research design, data collection, analysis, and interpretation. Within the panchayats, 20 clusters (individual villages or groups of habitations) of roughly similar size were selected randomly from two groups of habitations located on and off a main road, respectively.

The first household survey was conducted in September and October 2016, at the start of SBM implementation in the study area (as detailed in [40]). The second survey occurred from mid-October to early December 2019, immediately following the conclusion of the SBM program period. The same cluster-based sampling was used for both surveys and both samples are approximately proportional at the cluster level. However, they did not cover the identical sets of households so it can be said that repeated cross-sectional design was employed. A random walk method was used to sample households within the clusters. Household heads were interviewed and if not available another adult member was interviewed. Five and six trained enumerators collected the data in 2016 and 2019, respectively. Both surveys combined structured interviews (in Hindi) with direct observations of toilets and their surroundings. The 2016 survey was conducted in 499 households covering 2970 individuals, while the 2019 survey in 871 households covering 5037 individuals. This reflects the endline survey’s more extensive examination of various SBM implementation themes. In the 2016 survey, we used an interview schedule with 84 questions and direct observations to assess 17 parameters. In the 2019 survey, we replaced some less relevant questions and added approximately 20% new questions, mainly focused on experiences with SBM implementation and attitudes toward it.

In addition, our research in the study area contained a qualitative component that provided important insights into the processes and issues around SBM implementation. Due to space limitations, we use only a part of the qualitative data collected in 2019 and 2020 when we conducted 60 semi-structured interviews and 11 focus group discussions mostly but not exclusively with various SBM implementers from the grassroots to the state-level (specification can be found in Supplementary materials S1). Only some summarized findings derived from analysed qualitative data are presented in this article. The interviews covered a wider range of topics which differed based on the types and positions of interviewees, while addressing two general thematic areas. The first covered various topics around the current and past sanitation situation, behaviours, and attitudes in the study area discussed within the nexus of sanitation, hygiene, water, and development. The second thematic area addressed various issues around SBM implementation such as the organization of the SBM at different levels (from the central and state-level to the level of individual panchayats and communities), institutional support and background, financial resources and flows, training activities, and, in particular, implementation at the ground level.

### Ethics

All participants and informants participated in the study voluntarily and were assured of anonymity and confidentiality. Free and informed consent was obtained orally as we believe that asking for written consent would have made respondents uncomfortable. The project was approved by the institutional ethics committee of Charles University [approval numbers 2015/32 and 2019/16].

### Measures and analysis

In addition to the presentation of various descriptive findings , we use binary logistic regressions to model relationships between the structural and sanitation-related psychosocial variables (considered as independent variables) and toilet adoption as the dependent variable. Toilet adoption is constructed as a dichotomous measure distinguishing those households who owned functional toilets and reported their consistent use as differentiated from the rest of the households in our sample. A functional toilet was defined as a sanitation facility that was usable at the time of our survey, which means not blocked, broken, or missing basic components necessary for its use. Consistent toilet use was measured through a battery of questions on the defecation practices of respondents and household members in rainy and dry seasons. Explanations of the structural and psychosocial variables used in the regression analyses together with their basic descriptive statistics appear in Supplementary material S2 and S3.

Regression analyses were conducted separately for the 2016 and 2019 data to allow comparisons of the roles of individual variables and changes in their roles before and after SBM implementation. In a first step, we analysed the effects of structural variables to examine whether and how SBM implementation eliminated structural inequalities in toilet adoption. In a second step, we estimated the effects of individual psychosocial variables by adding them separately to the regression model containing the statistically significant structural variables from the first step.

## Findings

### Process of SBM implementation

The SBM received strong political support from the Indian Prime Minister and bureaucratic support for it was also visible at the state level in Jharkhand. During the period of the implementation of the SBM’s first phase in Jharkhand, the ruling party (for which the SBM was a flagship program) at the centre and the state level were the same, and this can be argued to be a reason for the strong bureaucratic backing that the program was seen to receive in this context. The SBM was implemented by the Jharkhand Drinking Water and Sanitation Department and bureaucratic support for it was apparent in our interviews with state-level senior bureaucrats as well as district- and block-level SBM officers.

At the village-level in Jharkhand, Village Water and Sanitation Committees were responsible for SBM implementation under the supervision of district- and block-level officers. The committees consisted of multiple members of elected and nominated local representatives, who underwent training for SBM implementation. Amongst these representatives, Mukhiyas (village heads) and Jal Sahiyas (translated as water helpers) can be highlighted since they played an important role in SBM implementation. Jal Sahiyas are exclusively females nominated to their positions by their communities and appointed by the Jharkhand Drinking Water and Sanitation Department. It is important to note that before the implementation of the SBM their role was a technical one– they would be given water testing kits and would be tasked with testing local water quality. After the start of the implementation of the SBM their technical role increased in complexity since they were tasked with organizing the construction of SBM toilets and, in some cases, they also worked as masons to construct these toilets. In addition, they were also tasked with sanitation behaviour change communication, as well as monitoring and even enforcing usage of SBM toilets. As our interviews revealed, their SBM-related training had been primarily focused on toilet construction and its monitoring, and enforcing toilet usage. They were nevertheless not given training in the provision of information on sanitation and hygiene.

Our interviews uncovered that Jal Sahiyas were generally proud of their extensive work for SBM implementation and had enjoyed their roles as sanitation-change agents but complained about their inadequate financial remuneration. Mukhiyas had been elected to their positions at the end of 2015– their official mandate relates to acting as an intermediary between their electorate and the local rural administration. Since SBM implementation began soon after they were elected, Mukhiyas expressed that implementing this program had become the main focus of their work. The prior experience and mandates of these two key local-level SBM implementers is foregrounded since it impacts on the manner in which the SBM was implemented in Jharkhand.

The SBM provided subsidized toilets for individual rural households at a cost of Rs. 12,000 (127 USD). A large part of Mukhiyas and Jal Sahiyas’ initial SBM-related work consisted in conducting surveys to determine who was eligible to receive such a subsidized toilet. According to official SBM records, construction of the SBM toilets in the study area started in 2016 with 50% constructed in 2016-17, 11% in 2017-18 and 32% in 2018-19 and our surveys confirmed this pattern. A minor share of households (5%) in the 2019 sample were reportedly excluded from the SBM. They explained that their names were not in the list of SBM beneficiaries or that information about SBM support had not reached them. However, the excluded households were largely concentrated in a few specific panchayats.

Nearly all (97%) SBM constructed facilities visited in the 2019 household survey were pour-flush toilets with two pits, uniform in design. Almost all (98%) of the 733 interviewed SBM beneficiaries confirmed that they contributed to the construction of these facilities and 92% asserted that this contribution was obligatory. All but six of these households reported contribution by labour, mostly but not solely by digging the pits. In addition, 18% of them reported material contributions. It was reported by several Jal Sahiyas and Mukhiyas that some beneficiaries had needed to be forced to contribute their labour. However, as per 2019 survey findings, 83% of respondents said that they had not minded their contributions to toilet construction.

In addition to the toilet construction subsidy, in Jharkhand, Rs. 765 (11 USD) per toilet was additionally earmarked for administration, behaviour change communication activities, geotagging of toilets, etc. In some cases, Jal Sahiyas were involved in geotagging toilets, but their main role lay in monitoring and enforcing toilet usage. Jal Sahiyas and Mukhiyas did talk about their efforts to verbally convince SBM beneficiaries of the need to use their toilets. Some of the more coercive tactics that were also mentioned involved threats of fines/withdrawal of government benefits, or the shaming of OD practice. Similarly, a minority of 7% of respondents recalled the use of punitive coercive measures (i.e., other than verbal warnings and explanatory convincing) and 4% reported that they were threatened or embarrassed in relation to SBM implementation.

Jal Sahiyas, Mukhiyas and other local SBM implementers were also involved in other behaviour change strategies involving mobilizing school children to spearhead sanitation behaviour change. However, for sanitation behaviour change communication, principally, activities and events were conducted by SBM teams that were deployed from the district level to villages– this was described in interviews by SBM implementers from the district level. These activities and events consisted of community meetings, workshops, street plays, or mural art communicating sanitation messages. According to the 2019 survey findings, the exposure of SBM beneficiaries to such behaviour-change activities was rather moderate. Only around one-third of respondents remembered that at least one information promotion or behaviour-change activity had been organized in their village during SBM implementation and slightly less than one-third reported participation in these activities. The reported participation was mostly in community meetings focused on hygiene and sanitation (23% of respondents reported participation) and toilet construction workshops (6%). Other events, such as street-plays, wall-paintings, transect walks, and village mapping, were mentioned solely on an anecdotal basis.

It should be noted that at the village level in Jharkhand, Swasth Sahiyas who work under the Department of Health, and maternal and child welfare workers called Anganwadi Sevikas who work under the Department of Women, Child Development and Social Security, have long been involved and trained in sanitation behaviour change communication (in each panchayat, a Swasth Sahiya and an Anganwadi Sevika were interviewed). However, we found that the SBM had not liaised with these village-level workers and volunteers since they did not work under the jurisdiction of the Jharkhand Drinking Water and Sanitation Department.

### Effects of the SBM on the change in sanitation conditions

The stated objective of the Swachh Bharat Mission (SBM) was to achieve an OD-free status in India by 2 October 2019, with a focus on expanding toilet coverage and promoting toilet usage as the ultimate goals. According to official SBM data, within the panchayats under our research, the baseline toilet coverage was only 7%, but it reached 100% by 2019 (as shown in Supplementary material S4).

**Fig. 3:**
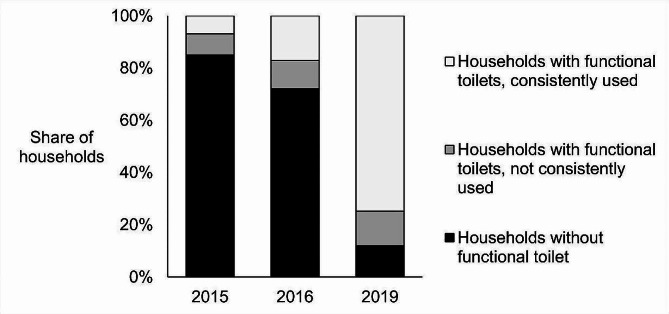
Change in toilet coverage and use before and after SBM implementation

Figure 3 illustrates our findings regarding the changes in toilet coverage and usage. Our data from 2016 revealed that 15% of households in our sample already had functional (usable) toilets before the SBM’s implementation in 2015. By October 2016, this figure increased to 28%. In our 2019 survey, we identified an 88% toilet coverage rate. Notably, approximately 98% of households that adopted toilets between 2015 and 2019 indicated that they were constructed under the SBM. This implies that around 71% of households in the 2019 sample received their toilets through the SBM, while 8% had non-SBM toilets, primarily obtained before the mission. This observation aligns with the aforementioned 15% baseline coverage from our 2016 data, suggesting that some households acquired new SBM toilets even if they already had a functional one.

In 2016, only 54% of households with functional toilets reported consistent usage, but this percentage increased to 85% in the 2019 sample. When considering the toilet coverage data mentioned earlier, it becomes evident that approximately 26% of households in the study still practiced open defecation either regularly or seasonally immediately after the completion of SBM implementation (as of our 2019 endline survey).

This trend aligns with respondents’ opinions on the sanitation behaviour of others in their villages. About 65% of respondents stated that a minority of others primarily practice open defecation, and 18% mentioned that the majority of people in their villages do the same.

A minority of respondents in the 2019 survey (13%) explicitly admitted their preference for OD over toilet use. This was the case for 20% of those without a functional toilet, while 80% of these households denied such preference. Two-thirds of them explained the absence of a toilet by referring to a lack of space, to the fact that they were a recently settled family or did not provide any explanation. The rest of those without toilets, which corresponded to 5% of the 2019 sample, were those reportedly excluded from the SBM.

Of families with toilets and children below five, 31% and 35% reported safe disposal of children’s faeces into a toilet in 2016 and 2019, respectively. According to the endline survey, a majority (69%) of families with children under five, who were provided with toilets under SBM, did not report using these toilets for the disposal of their children’s faeces. Instead, the most common practice reported by 55% of these families was to discard it in open areas. If we consider burning of children’s faeces also as a safe practice, 20% and 39% of all families (including those without toilets) with children under five safely managed children’s faeces in the 2016 and 2019 samples, respectively. Although no less essential than the management of adults’ faeces, these results indicate that the safe management of children’s faeces received much less attention in SBM implementation.

Comparison of SBM and non-SBM toilets in Table 1 offers another perspective on the sanitation change induced by the SBM. It assesses quality standards of newly introduced SBM facilities and their convenience for users relative to the non-SBM toilets. It considers solely functional (usable) facilities, while the sample contained also 40 additional SBM toilets (5% of all SBM facilities) that were not functional (blocked, broken, or not completed). Unlike for the subgroup of SBM toilets, all surveyed non-SBM toilets were functional and they also less often revealed apparent technical deficiencies. Almost all households with non-SBM toilets (99%) reported their consistent use, while this was true for 84% of households with SBM toilets. The most non-SBM toilets were built by households themselves, based on household demand. These toilets had also been built either in or adjacent to a house and were provided with piped water– showing that sanitation infrastructure had been built in a way to support toilet usage. It was also mentioned by Jal Sahiyas that some of the identified SBM beneficiaries who did not express demand for toilets wanted them to be built far away from their houses. Unlike non-SBM facilities, SBM toilets contained water tanks attached to the toilet from outside with an outlet tap inside. These tanks were nevertheless very rarely used.

**Table 1:** Comparison of SBM and non-SBM functional toilets at the time of our endline survey at the end of 2019 (after the SBM implementation)

	All toilets	SBM toilets	Non-SBM toilets
Number of functional (usable) toilets	761	692	69
Years elapsed from toilet construction (average)	2.1	1.5	8.2
Toilets without apparent technical deficiencies (working water seal, slab, roof, doors, walls)	72%	70%	87%
Consistently used toilets	85%	84%	99%
Well managed and clean toilets	90%	88%	96%
Distance of toilets from house (average in meters)	7.7	8.2	1.8
Toilets located in or directly at house	39%	37%	62%
Toilets connected to piped water	11%	5%	51%
Toilets with water available at the time of survey	71%	68%	86%
Toilets with soap available	42%	39%	64%

### Contextual drivers

#### The role of structural constraints

Table 2 reports results on the relationships between structural variables (demographic, socioeconomic, and sociocultural characteristics) and toilet adoption (ownership of functional and consistently used toilets). Regression estimates are reported for two separate models based on the 2016 and 2019 data. The results based on the 2016 data document that inequality in toilet adoption was associated with the differences in socioeconomic or sociocultural characteristics. It implies an existence of structural sanitation inequalities related to differences in income, attained education, and religion (see also [40]). The results obtained from the 2019 data are quite different. The overall model fit was considerably weaker and none of the analysed structural variables was a statistically significant predictor of toilet adoption after the SBM. A notable positive effect (i.e., higher toilet adoption) was found for Muslim families when compared to Sarna (nature religion practised by groups that claim an indigenous status) households but even this relationship was not statistically significant.

**Table 2:** Demographic, socioeconomic, and sociocultural variables, and their relationships with toilet adoption (beta coefficients and standard errors estimated by the binary logistic regressions with the ownership of functional and consistently used toilets considered as the dependent variable)

	2016 (before SBM)			2019 (after SBM)		
	Representation in the sample	Beta coefficient	Standard errors	Representation in the sample	Beta coefficient	Standard errors
If female respondent	56%	-0.059	0.296	45%	-0.140	0.154
Age of respondent	32.57	0.010	0.017	35.55	0.000	0.000
Household size	5.95	-0.071	0.066	5.78	0.017	0.034
Religion:						
Hindu	59%	0.713	0.658	57%	0.024	0.156
Muslim	8%	2.285	0.832*	8%	1.012	0.556
Christian	5%	2.421	0.591**	6%	0.014	0.555
Sarna	28%	Reference category		28%	Reference category	
Education:						
No	32%	-1.109	0.524*	38%	-0.439	0.321
Up to lower secondary	37%	-1.037	0.323**	39%	-0.229	0.264
Higher secondary	18%	-0.846	0.308*	14%	-0.066	0.336
College	13%	Reference category		9%	Reference category	
Main source of livelihood:						
Farming	22%	1.237	0.651	29%	-0.304	0.179
Self-employed	16%	0.919	0.568	13%	0.545	0.273
Other	15%	1.008	0.448*	13%	-0.325	0.224
Casual labour	47%	Reference category		45%	Reference category	
Household income (in logarithms)	0.00	1.653	0.376**	-0.05	0.025	0.396
Size of owned land (in logarithms)	-0.65	-0.039	0.145	-0.55	0.028	0.110
Nagelkerke (Cox and Snell) R^2^		0.25 (0.15)			0.04 (0.03)	
N	481			871		

*Notes* * Statistically significant at the 0.05 level, ** at the 0.01 level. Accounted for data clustering. Household income normalized by the median income of a respective year. Other variables such as the presence of children below five in households, presence of elderly people in households, sex of household head, and social category (SC/ST/OBC/Other) were also examined, but none of them was statistically significant

#### The role of psychosocial variables

Table 3 shows changes in the psychosocial variables between 2016 and 2019 and the statistical relationships of these variables with the toilet adoption before and after the SBM. Let us recall that regression estimates for individual variables were obtained by including them separately into the regression model in Table 2 to account for possible confounding. In addition, Table 4 presents analogous regression estimates for few other thematically relevant psychosocial variables that were measured only in the 2019 survey. We can see that several of the considered psychosocial variables were found to be statistically significant correlates of toilet adoption. This is especially true for the set of results pertaining to data collected in 2019.

The last column of Table 3 reveals that one of the most pronounced changes was observed for the satisfaction with one’s own sanitation situation– this increased by 57%. The reported satisfaction was closely associated with toilet adoption in both pre-SBM and post-SBM data sets. That the expansion of access to toilets moved the satisfaction up once again documents a strong preference for toilet use over OD practice.

Similar observation applies to the perception of descriptive norms related to toilet use that also went up significantly hand in hand with expanding access to toilets and their use. The share of those who stated that the majority of other people in the village mostly defecate in a toilet increased by 69% from 6% in 2016 to 75% in 2019. Interestingly, these figures correspond almost exactly to the toilet adoption rates reported above   The perception of injunctive norms was strong already at the beginning of the SBM and increased slightly further with SBM implementation. It suggests an existence of social pressure on (and surveillance of) toilet use. This can be linked to findings in Table 4 on a significant negative regression coefficient obtained for a measure of social sanctions (question on what would happen to those who are spotted when practicing OD) and a significant positive coefficient obtained for a measure of social capital (question on how people in the village work together towards the common goal of making it clean).

The level of sanitation-related knowledge in our sample was rather low in 2016 and increased only marginally during SBM implementation. It may be related to the already mentioned limited exposure of local people to SBM awareness and information promotion activities. This is further confirmed by the results in Table 4 showing that only 31% of respondents recalled any such activities organized in their villages, while only 21% of them attended a village meeting that addressed hygiene and sanitation behaviour. At the same time, the relationship between exposure to these activities and toilet adoption was significant and positive confirming its importance for successful sanitation change. Moreover, in the regression analyses reported in Tables 3 and 4, all of the variables that measured sanitation and hygiene-related knowledge revealed statistically significant relationships with toilet adoption.

Willingness to pay for SBM-like toilets was a slightly lower in 2019 compared to 2016 and the opposite holds true for the measure of unwillingness to pay, which increased by 10% (Table 3). These results are not surprising and may be linked to the role of subsidies in the SBM. In addition, willingness to pay was positively related (and the unwillingness negatively related) to toilet adoption after SBM implementation.

In both surveys, around half of respondents mentioned positive health benefits among reported toilet advantages. Perception of health risks associated with OD was also similar for both data sets. The stability of health-related perceptions can be compared to the perceptions of non-health benefits of toilets such as privacy, comfort, easy access, or safety that increased considerably more (by 19% for safety, 24% for safety, 33% for comfort, and 40% for easy access). It seems that the expanded toilet availability and use impacted more the perception of sanitation non-health benefits, which are easier to recognize based on personal experience, than the perception of health benefits of toilets, that are not immediate.

Shortages of water were perceived as a comparatively less serious problem by households in 2019 than in 2016 (Table 3). It may indicate that the expansion of toilet coverage didn’t heighten general concerns about water shortages in the study area. However, unlike in the 2016 survey, the problem of water shortages was significantly more accentuated by those who didn’t have or didn’t use toilets after the end of the SBM implementation in 2019 (at the time of our endline survey). The latter subgroups also revealed higher concerns about difficulties associated with securing water for toilet use (Table 4). Therefore, water-related constraints (whether objective or perceived) still represent a consequential barrier for making the study area OD-free.

**Table 3:** Psychosocial measures, their change between 2016 and 2019, and their relationships to the ownership of functional and consistently used toilets

	2016 (before SBM)			2019 (after SBM)			Change 2016–2019
	Representation in the sample	Beta coefficient	Standard errors	Representation in the sample	Beta coefficient	Standard errors	
Satisfied with current sanitation practice	31%	4.798	0.548**	88%	3.712	0.379**	+ 57%
Willing to pay Rs. 12,000 for SBM-like toilet	46%	0.831	0.446	45%	0.647	0.216**	-1%
Not willing to pay anything for SBM-like toilet	21%	0.171	0.636	31%	-0.954	0.266**	+ 10%
Health benefits acknowledged among toilet advantages	52%	-0.299	0.340	50%	0.995	0.189**	-2%
OD perceived as risk for health	63%	-0.222	0.265	66%	0.754	0.220**	+ 3%
Privacy acknowledged among toilet advantages	51%	0.611	0.353	70%	0.514	0.155**	+ 19%
Comfort acknowledged among toilet advantages	53%	0.082	0.344	86%	0.619	0.269*	+ 33%
Easy access acknowledged among toilet advantages	38%	0.199	0.330	78%	0.294	0.205	+ 40%
Safety acknowledged among toilet advantages	17%	-0.270	0.520	41%	-0.022	0.168	+ 24%
Diarrhoea outbreaks reported among serious threats	50%	-0.136	0.453	17%	0.084	0.220	-33%
Water shortages reported among serious threats	34%	0.600	0.390	28%	-0.666	0.219**	-6%
At least some knowledge on diarrhoea prevention	15%	0.772	0.324*	20%	0.832	0.247**	+ 5%
Proper use of toilet among recalled health and sanitation messages	21%	1.014	0.268**	30%	0.646	0.189**	+ 9%
Proper management of child faeces among recalled health and sanitation messages	1%	0.014	1.010	8%	0.783	0.373*	+ 7%
Stated that majority of other people in his/her village mostly defecate in toilet	6%	1.559	0.516**	75%	0.988	0.187**	+ 69%
Stated that all other people should defecate in toilet	84%	0.371	0.498	87%	0.810	0.194**	+ 3%
Agreed that people in the village think that he/she should defecate in toilet	82%	-0.957	0.386*	90%	0.661	0.259*	+ 8%
N	481			871			

*Notes* * Statistically significant at the 0.05 level, ** at the 0.01 level. Based on the binary logistic regressions. Accounted for data clustering. Beta coefficients and standard errors obtained from the binary logistic regression when individual psychosocial predictors separately added into the regression model specified in Table 2

**Table 4:** SBM-related psychosocial variables measured in 2019 only and their relationships to the ownership of functional and consistently used toilets

	Representation in the sample	Beta coefficient	Standard errors
Remembered sanitation-related activities organized in her/his village during SBM	31%	0.576	0.184**
Attended village meeting about hygiene and sanitation behaviour during SBM implementation	22%	0.742	0.166**
Knew how does double-pit system worked	53%	0.895	0.193**
Planned to reuse pit content as fertilizer	32%	0.810	0.215**
Was unhappy about contributing so much time and energy to toilet construction	16%	-1.083	0.274**
Would prefer OD if not forced to use toilet	13%	-1.327	0.235**
Found difficult to get water for toilet	24%	-1.249	0.245**
Stated that nothing happens to people who are spotted when practicing OD in her/his village	33%	-0.333	0.148*
Agreed that majority of people in her/his village work together towards the common goal of making the village clean	14%	1.243	0.347**

*Notes* * Statistically significant at the 0.05 level, ** at the 0.01 level. Based on the binary logistic regressions. Accounted for data clustering. Beta coefficients and standard errors obtained from the binary logistic regression when individual psychosocial predictors separately added into the regression model specified in Table 2

## Discussion

Local case studies designed to comprehensively examine the intricacies of sanitation interventions by simultaneously exploring the implementation process, changes in outcomes, and the role of contextual drivers can make an irreplaceable contribution to the evidential diversity of the performance of programmes such SBM. To our best knowledge, the published evidence on the Indian SBM represents a notable example of the absence of such studies. Although large-scale surveys and a few case studies documented that the SBM has not achieved its goal to eliminate OD practice in India [14, 16], the extent and nature of sanitation change induced by the SBM in specific local contexts are poorly understood. This motivated the present study which has sought to understand sanitation change induced by SBM in rural Jharkhand by pursuing the POC (process-outcomes-context) approach.

The SBM Implementation in Jharkhand gained political support and bureaucratic commitment. However, our study indicated a lack of convergence between the involvement of different departments contradicting calls for the use of ‘whole-of-government’ approaches [54, 55]. More specifically, the Jharkhand Drinking Water and Sanitation Department was responsible for SBM implementation. Unlike the Jharkhand Health Department or the Jharkhand Department of Women, Child Development and Social Security, its grassroots-level volunteers had no prior experience of sanitation behaviour change communication and had worked in a technical capacity till the start of the SBM. Moreover, their SBM-related training also did not have a strong sanitation behaviour change focus, though the latter was clearly emphasized in the official SBM guidelines [56]. It may be linked to our observation that the behaviour-change activities were sidelined during the ground-level implementation in the study area that primarily concerned with the construction of toilets. For example, the rates of attendance and recalls of these activities were substantially lower than reported for another sanitation intervention in Odisha that had an explicit behaviour-change focus [24]. The neglect of a behaviour-change component was criticized with respect to the previous Indian sanitation campaigns [6, 57]. Our findings suggest that this problem was not eliminated in the SBM implementation in the study area (though the awareness of SBM was considerably more bolstered by its extensive coverage in media (e.g., [58]).

Our findings showed that the SBM efforts and resources were largely focused on the provision of subsidized toilets. Their construction was conditional upon obligatory labour contribution from beneficiary households. SBM beneficiaries mostly did not contest this requirement and SBM implementation more generally. Antagonistic attitudes were identified in a minority of around 5% of households, mostly because they were or felt excluded. These cases were concentrated in a few specific panchayats. Otherwise, our study uncovered a relatively good acceptance of SBM in the study area.

The results of this study showed that the share of households owning functional toilets increased from 15% to 88% in the course of SBM implementation between 2015 and the endline survey in 2019. Almost all (98%) households who adopted toilets in this period confirmed that they got them from the SBM. Some of them may have adopted toilets even if SBM implementation had not occurred so the exact (hypothetical) effect of the SBM on toilet coverage remains unknown. Given the sluggish sanitation dynamics prior to the SBM, it is nevertheless quite certain that the SBM attributes for a great deal of the observed change in the functional toilet ownership rate. When additionally considering our findings on self-reported toilet use, we found that 74% of households owned and consistently used toilets, while 26% of them practiced OD on a regular or a seasonal basis in the study area at the time of our endline survey in 2019.

Based on the NFHS-5 2020/21 data, a slightly lower OD rate of 23% and a considerably higher OD rate of 49% were reported for Ranchi district and rural Jharkhand, respectively [14]. However, the estimate for Ranchi district was determined from a sample that included not only rural but also urban households that tend to have higher sanitation rates. It is thus very likely that, compared to the averages pertaining to both rural Ranchi district and rural Jharkhand, our study area represents a region with a better sanitation situation and also better SBM performance. A plausible explanation is the proximity of our study area to the state capital because both physical and institutional remoteness tend to impact sanitation negatively [40, 59].

Earlier research documented the revealed preference for OD practice in India explained by the reluctance to use low quality or inconvenient toilets provided under previous sanitation programmes [60, 61], though more recent research suggested the primary role of economic and ecological constraints [62]. Findings on stated preferences for OD elicited in our survey do not indicate that the former argument applies in the present context as only a small minority of respondents reported a preference for OD. However, the long-term sustainability of the observed sanitation change remains a key question. A significant share of respondents (31%) expressed unwillingness to invest in SBM-like toilets and it may affect their willingness to maintain these facilities and invest in any necessary repairs and manage pit contents. Moreover, the comparison of SBM and non-SBM sanitation facilities in our sample demonstrated that the SBM toilets had inferior quality standards and provided lower comfort for users. It heightens a risk of gradual slippage back towards practicing OD.

Of the differences revealed in the comparison between SBM toilets and non-SBM facilities, the two most significant were the lack of piped water in the vast majority of SBM toilets and their poorer accessibility. These disparities raise doubts about the sustainability of SBM toilet utilization, a concern that has been emphasized previously [23, 24, 63]. The former problem may at least partly be addressed by the ongoing Jal Jeevan Mission that plans to link all households to piped water. However, the prospects are uncertain, and Jharkhand belongs to the states with the most sluggish progress [64]. Realistically, it can be expected that a non-negligible proportion of SBM toilets will sooner or later remain unused. Follow-up monitoring and measures to minimize OD slippage is required.

We found that structural factors, such as inequalities in income, education, and religion, explained the variation in toilet adoption in the study area before the SBM (see also [40]). This included lower sanitation rates among Sarna households, nearly all of which belonged to the ST (scheduled tribes) social category. Importantly, our study confirmed that SBM implementation effectively eliminated these structural sanitation inequalities, at least in a statistical sense. After the SBM concluded, structural factors were no longer statistically significant predictors of toilet adoption in the study area. The finding that the SBM reduced structural constraints for toilet adoption in the surveyed region is significant, as emphasized by prior studies [10, 62], though we noted above that this did not hold for persisting ecological constraints related to water availability.

However, our research reveals that the SBM was less successful in addressing the psychosocial determinants of safe sanitation. Psychosocial factors emerged as strong predictors of the remaining variation in toilet adoption in our endline survey data after the SBM’s conclusion. This finding underscores the continued importance of effective sanitation-related education and behaviour-change efforts. At the same time, our results offer a few more nuanced suggestions regarding this. We saw that both descriptive and injunctive social norms around toilet use have already been established. Thanks to the wide coverage of the SBM and its massive propagation in the media, there is a widespread awareness that toilet use is recommended and required (both administratively and socially). However, a persisting gap was identified with respect to the actual understanding and knowledge about safe sanitation and hygiene practices and the implementation of the SBM didn’t bring adequate change in this respect.

A notable example of overlooked awareness pertains to the safe disposal of child faeces which continues to be low in the study area after the end of SBM. This aspect of hygienic behaviour was neither adequately integrated into the SBM guidelines nor specifically addressed during SBM implementation in the surveyed region. As a result, the level of knowledge about this was generally weak both at the beginning and the end of SBM implementation. The safe disposal in households with small children increased modestly from 20 to 39%, if burying is considered a safe practice. Of those with small children and SBM toilets, only less than one-third reported using these facilities for the disposal of their children’s faeces. The safe management of child faeces remains uncommon in India [24, 65] and has been generally neglected [66]. The Indian government should address this gap by more effectively integrating this aspect into health and sanitation interventions [24, 67].

That the SBM did not have a large impact on improving sanitation-related knowledge is notable and it may also have been caused by the above-mentioned low attendance at the village meetings that addressed hygiene and sanitation behaviour which had been organized by district-level SBM teams. It can also be noted that Jharkhand is a context in which witch-doctors/herbalists are approached for disease cures and health knowledge– as was found by our fieldwork. Although it is a demanding and perhaps arduous task, education and information promotion focused on providing substantive information to facilitate real understandings of the importance of safe sanitation remains a key challenge that was neglected during SBM implementation.

Certain households obtained new SBM toilets even though they already had functional ones. This could be attributed to factors like household size or multiple generations cohabiting. Another reason cited was that the new SBM toilet would be necessary soon. The observation may nevertheless also point to problems in SBM implementation.

This article has undoubtedly some limitations. First, the study was deliberately designed to focus on multiple domains of SBM performance (i.e., process, outcomes, context). It results in a wide thematic scope which necessarily comes at the expense of the depth of information provided on individual domains. Although each of them might have been possibly analysed in more detail separately, it was the core intention to cover them simultaneously within a single paper. The slicing of research findings (in our opinion a common practice in sanitation research) may overlook interdependencies between findings on particular domains and, eventually, disintegrates the picture that this local study attempts to paint. Secondly, we applied a rather narrow definition of contextual factors by considering the measurable situational characteristics of individuals, households, or communities. Although this pragmatic conceptualisation allowed us to quantitatively examine how their role changed over the course of SBM implementation, it can only partly capture the influence of wider political, sociocultural, and environmental contexts. Thirdly, toilet usage and other aspects of sanitation behaviour were examined based on self-reported information and may be subject to social desirability bias. The relevance of the estimated rates of toilet usage was nevertheless corroborated based on the question on toilet usage of others (descriptive norm). However, similar checks were not possible for other findings. For example, the evaluation of the SBM in household surveys as well qualitative interviews may also be prone to this bias, particularly if considering the politicization of the SBM and its prominence in the media.

## Conclusion

This article examined the extent and nature of the sanitation change induced by the Indian SBM in rural Jharkhand. We identified political support of the SBM implementation and its acceptance amongst the population. Female community workers became key agents of SBM implementation at local level. The SBM primarily concentrated on meeting targets regarding the construction of subsidized toilets. The behaviour change component was underplayed, focusing more on spreading normative sanitation messages and less on public education. Between 2015 and 2019, the SBM increased toilet coverage from 15% to 85% and reduced the OD rate in the study area to 26%. It curbed structural sanitation inequalities in access to functional toilets, furthered social sanitation norms, improved attitudes towards toilet use, but only negligibly impacted on hygiene and sanitation awareness. Sustainability of the observed sanitation change is uncertain and remains a key question for future research. Gradual return of at least some people to OD practice is likely due to challenges related to water unavailability, unwillingness to maintain toilets without further subsidies, or insufficient user comfort of the SBM toilets. Secondary data suggests that the performance of the SBM in the selected study area was better than in the majority of other parts of Jharkhand. This article calls for more systematic production of contextually specific knowledge on the performance of sanitation interventions.

### Electronic supplementary material

Below is the link to the electronic supplementary material.

Supplementary Material 1

## Data Availability

Primary data are available from the corresponding author based on a reasonable request.

## Abbreviations

NFHS: National Family Health Survey
NGO: Non–Governmental Organization
OBC: Other Backward Classes
OD: Open Defecation
POC: Process, Outcomes, Context
SBM: Swachh Bharat Mission
SC: Scheduled Classes
ST: Scheduled Tribes
