# Supplementary material for: The process, outcomes and context of the sanitation change induced by the Swachh Bharat Mission in rural Jharkhand, India
Source: BMC Public Health. 2024 Apr 12;24:997. doi: 10.1186/s12889-024-18388-y (PMC11015623; doi:10.1186/s12889-024-18388-y)
Supplement: Supplementary file 1 — Supplementary Material 1 [file 12889_2024_18388_MOESM1_ESM.pdf]

SUPPLEMENTARY MATERIALS

Supplementary material S1: Information on semi-structured interviews and FDGs

Type of interviewees/FGD participants	Description of position	Number of interviews	Notes
Jal Sahiyas	Community WASH change agents	13 interviews + 2 FGDs	All females. FGDs conducted with groups of 18 and 2 participants. Includes 1 pre-test interview. Plus 1 follow-up interview.
Mukhiyas	Panchayat headpersons	15 interviews	11 females and 9 males. Includes 3 pre-test interviews. Plus 2 follow-up interviews.
Rural Health Workers	Community childcare workers, Community health volunteers, Traditional healers	12 interviews + 4 FGDs	10 females and 2 males. FGDs with groups of 2, 3, 3, and 9 participants
Other interviewees/FGD participants at village and Panchayat level	Villagers in various positions not directly related to WASH	9 interviews + 2 FDGs	7 males and 2 females. FGDs with groups of 4 and 4 participants. Plus 1 follow-up interview.
Higher level SBM Implementers	Sanitation experts and government SBM staff from the block to state levels	11 interviews + 3 FGDs	11 males and 1 female. FGDs with groups of 2, 2 and 6 participants. Plus 3 follow-up interviews.

Supplementary material S2: Structural variables examined in the regression analyses with their basic descriptive statistics

	2016	2019
Demographic characteristics		
Age of respondent – mean age (SD)	32.57 (11.89)	35.55 (13.26)
Share of female respondents	0.56	0.46
Share of female household heads	0.13	0.19
Household size – mean size (SD)	5.96 (2.54)	5.78 (2.03)
Share of households with elderly (above 50) members	0.51	0.58
Share of households with children (under 5)	0.38	0.39
Share of households with disabled people	0.06	0.08
Sociocultural variables		
Religion (relative shares):		
Hindu	0.59	0.57
Muslim	0.07	0.08
Christian	0.05	0.06

Sarna	0.29	0.29
Social category (relative shares):		
Scheduled castes	0.07	0.06
Scheduled tribes	0.45	0.49
Other backward classes	0.44	0.40
Other	0.04	0.05
<u>Socioeconomic variables</u>		
Attained education of respondent; of household head; higher of household's head or respondent (relative shares):		
No	0.32; 0.36; 0.20	0.38; 0.48; 0.29
Up to lower secondary	0.38; 0.51; 0.47	0.40; 0.42; 0.46
Higher secondary	0.18; 0.07; 0.19	0.13; 0.06; 0.14
College	0.13; 0.06; 0.14	0.09; 0.04; 0.11
Annual household income in Rs. – median; mean (SD)	66000; 88802 (118286)	60000; 60660 (39352)
Land ownership in Acres – median; mean (SD)	050; 0.87 (1.47)	0.50; 0.92 (1.51)
Share of functionally landless	0.41	0.36
Primary source of households' livelihood – relative shares:		
Farming	0.22	0.29
Casual labour	0.48	0.45
Self-employment	0.15	0.13
Regular employment, private or public	0.15	0.13
Ownership of land in acres – median; mean (SD)	0.50; 0.87 (1.47)	0.50; 0.92 (1.52)
Type of house (relative shares)		
Mud-walled and similar	0.51	0.51
Semi-concrete or semi-mud-walled	0.29	0.33
Concrete	0.20	0.16
Change in economic situation over past two years (relative shares):		
Improved	0.41	0.28
Same	0.52	0.63
Worsened	0.07	0.08

Supplementary material S3: Psychosocial variables, their explanation, and basic descriptive statistics

	2016	2019
<u>Sanitation- and hygiene-related knowledge</u> *Knowledge of hygiene and sanitation messages* – asked as open questions and then classified into 10 predefined types of responses. Sum of relevant messages – range; average (SD):	0-6; 1.51 (1.66)	0-5; 0.83 (1.38)
Awareness about diarrhoea prevention – binary variable based on question to list the three most effective ways of preventing diarrhoea Share of those with at least some awareness:	0.15	0.20
<u>Perception of descriptive and injunctive social norms around toilet use</u>		
"Do other people in your village mostly use a toilet?" – All of them; Majority; Minority; None; Don't know	0.01; 0.05; 0.57; 0.23; 0.14	0.10; 0.65; 0.18; 0.01; 0.07
Do you think that other people in your village should defecate in a toilet? – All of them; Majority; Minority; None; Don't know	0.87; 0.00; 0.08; 0.03; 0.02	0.84; 0.11; 0.03; 0.00; 0.01

People in this village think you should use a toilet for defecation – Strongly agree; Agree; Disagree; Strongly disagree; Don't know	0.53; 0.30; 0.02; 0.01; 0.14	0.33; 0.58; 0.01; 0.00; 0.08
Do people in your village defecate in the open even though they have a toilet – All of them; Majority; Minority; None; Don't know	-	0.01; 0.10; 0.70; 0.11; 0.08
If you weren't forced to use the toilet, you would prefer open defecation – Strongly agree; Agree; Disagree; Strongly disagree; Don't know	-	0.01; 0.09; 0.21; 0.66; 0.03
Attitudinal variables<br>*Perceived advantages of toilet* – asked as open question and then classified into predefined types of responses. Used for constructing individual binary variables of perceived types of toilet advantages.<br>Sum of perceived advantages – average (SD)	2.67 (1.17)	4.16 (1.44)
Perceived disadvantages of OD – asked as open question and then classified into predefined types of responses. Used for constructing individual binary variables of perceived types of OD disadvantages.<br>Sum of perceived disadvantages – average (SD)	2.30 (1.12)	2.93 (1.36)
Satisfaction with current defecation practice – Average (SD) based on 5-poit scale: 1. Very satisfied; 2. Satisfied; 3. Neither satisfied nor dissatisfied; 4. Not satisfied; 5. Very dissatisfied<br>Toilet owners<br>Toilet non-owners<br>All respondents	 <br><br>1.85 (0.99)<br>4.17 (0.75)<br>3.50 (1.33)	 <br><br>1.53 (0.63)<br>3.64 (1.30)<br>1.79 (1.02)
Willingness to pay for toilet – constructed based on the following sequence of questions:<br>"Just imagine if you didn't have your toilet, would you pay at least 12,000 Rs. of your own money for a SBM type toilet?" … "If no, would you pay 6,000 Rs. of your own money for a SBM type toilet?" … "If no, how much would you pay for an SBM toilet (of your own money)?"<br>Distribution of responses:<br>If only for free<br>More than 0 Rs. but than 6000 Rs.<br>More than 6000 Rs. but less than 12000 Rs.<br>Willing to pay 12000 Rs. or more	0.22<br>0.13<br>0.17<br>0.48	0.31<br>0.06<br>0.18<br>0.45
Willingness to pay for toilet – constructed based on the following sequence of questions:<br>"Just imagine if you didn't have your toilet, would you pay at least 12,000 Rs. of your own money for a SBM type toilet?" … "If no, would you pay 6,000 Rs. of your own money for a SBM type toilet?" … "If no, how much would you pay for an SBM toilet (of your own money)?"<br>Distribution of responses:<br>If only for free<br>More than 0 Rs. but than 6000 Rs.<br>More than 6000 Rs. but less than 12000 Rs.<br>Willing to pay 12000 Rs. or more	0.22<br>0.13<br>0.17<br>0.48	0.31<br>0.06<br>0.18<br>0.45
Risk perception variables<br>*Perceived general threats* – question: "From the following list name the two most serious problems" Options: water scarcity, shortages of food, diarrheal disease outbreaks, malaria outbreaks, hikes in prices of basic commodities, losses of agricultural land or livestock.<br>*Perceived water stress* – share of those who selected water scarcity<br>*Diarrhoea perceived as a threat* – share of those who selected diarrheal disease outbreaks	0.34<br>0.50	0.28<br>0.17
OD perceived as risk for health – share of those who reported health risk among OD disadvantages	0.63	0.66

Supplementary material S4: Toilet construction under SBM in the surveyed Panchayats according to the SBM official data

	Baseline SBM survey	2015-16	2016-17	2017-18	2018-19
Households without toilets	9527	8908	4106	3025	0
Toilets constructed under SBM	0	619 (7%)	4802 (50%)	1081 (11%)	3025 (32%)

Note: According to the official SBM data, there were 9527 (93%) households without toilets and 1514 (7%) households with toilets before the SBM implementation in the surveyed panchayats. Source: SBM Target vs Achievement Report accessed on January 3, 2021, through:

https://sbm.gov.in/sbmReport/Report/Physical/SBM_TargetVsAchievementWithout1314.aspx
